# Review of biomarkers for response to immunotherapy in HNSCC microenvironment

**DOI:** 10.3389/fonc.2023.1037884

**Published:** 2023-02-13

**Authors:** Shaoshi Chen, Yifan Yang, Shizhi He, Meng Lian, Ru Wang, Jugao Fang

**Affiliations:** Department of Otorhinolaryngology Head and Neck Surgery, Beijing Tongren Hospital, Capital Medical University, Beijing, China

**Keywords:** head and neck squamous cell carcinoma, tumor microenvironment, PD-1, immunotherapy, biomarker, PD-L1

## Abstract

Head and neck squamous cell carcinoma are one of the most common types of cancer worldwide. Although a variety of treatment methods such as surgery, radiotherapy, chemotherapy, and targeted therapy are widely used in diagnosing and treating HNSCC, the survival prognosis of patients has not been significantly improved in the past decades. As an emerging treatment approach, immunotherapy has shown exciting therapeutic effects in R/M HNSCC. However, the current screening methods are still insufficient, and there is a significant need for reliable predictive biomarkers for personalized clinical management and new therapeutic strategies. This review summarized the application of immunotherapy in HNSCC, comprehensively analyzed the existing bioinformatic studies on immunotherapy in HNSCC, evaluated the current methods of tumor immune heterogeneity and immunotherapy, and aimed to screen molecular markers with potential predictive significance. Among them, PD-1 has obvious predictive relevance as the target of existing immune drugs. Clonal TMB is a potential biomarker for HNSCC immunotherapy. The other molecules, including IFN-γ, CXCL, CTLA-4, MTAP, SFR4/CPXM1/COL5A1, TILs, CAFs, exosomes, and peripheral blood indicators, may have suggestive significance for tumor immune microenvironment and prognosis of immunotherapy.

## 1 Introduction

Head and neck cancer has been the sixth most prevalent cancer worldwide, accounting for 880,000 new cases diagnosed. More than 450,000 deaths each year, according to a global cancer statistic in 2018 (1), with gender, HPV infection, tobacco, and alcohol as risk factors for patients (2–4). Over 90% of histological subtypes of head and neck cancer is squamous cell carcinoma, also known as head and neck squamous carcinoma (HNSCC), while 2% are sarcomas and the other 7% include melanomas, adenocarcinomas, and not well-specified tumors (5).

Unfortunately, the 5-year survival of HNSCC remained at 40-50% stably even with emerging treatments like immunotherapy in the past decades (6). Around 30% of patients would suffer from cancer relapse and treatment failure. Those high rates of relapse and metastasis remain the central issue of the treatment.

Immunotherapy has shown a powerful therapeutic effect in multiple cancers, such as non-cell lung cancer and melanoma. Common strategies include immune checkpoint inhibitors (ICI), tumor vaccination, dendritic cell immunotherapy (DC), antibody-drug conjugates (ADC), and adoptive T-cell transfer therapy (ACT). Those treatments based on cancer immunity have positive effects on prognosis. PD-1 inhibitor pembrolizumab and nivolumab were the only allowed ICI for recurrent/metastatic (R/M) HNSCC in the clinical, and the patients could get more therapeutic benefits from it, like longer OS compared with standard chemotherapy (7).

Progression-free survival (PFS), defined as the time to disease progression or death from any cause, is an essential short-term evaluation index for tumors and their treatment options. For patients with CPS < 1 and TPS < 50%, PFS was shorter in those with pembrolizumab than in those with standard care. However, multiple clinical trials have shown that PD-L1 inhibitors were hard to prolong PFS compared with standard chemotherapy (7–10).

PD-L1 inhibitors can induce adverse autoimmune reactions, known as immune-related adverse reactions (irAEs), mainly characterized by immune T-cell infiltration in various organs. Such adverse reactions can significantly affect patients’ quality of life and survival time. Patients who received pembrolizumab monotherapy had fewer treatment-related adverse events of grade 3 or more than those who received standard chemotherapy (55% vs. 83%). Moreover, pembrolizumab plus chemotherapy did not significantly increase the risk of adverse events (85%) (11). The most common adverse events of PD-1 inhibitors are hypothyroidism, fatigue, and anemia, among which hypothyroidism is relatively specific in immunotherapy (8, 9). Moreover, there was no significant correlation between the incidence of adverse immunotherapy events and the expression of PD-1 (9). Of the 246 patients enrolled, only four adverse events occurred in grades 3-5, including one in grade 5. This death was due to a severe inflammatory skin reaction known as Stevens-Johnson syndrome. Thus, pembrolizumab therapy has a favorable safety profile, and PD-1 inhibitors do not increase the risk of chemotherapy-related adverse events.

However, only 18% of patients with R/M HNSCC benefited from ICI (10). Hence, there must be a complex molecular interaction controlling the oncogenesis and interrupting it could be the key to treatment. ICI aims to reactivate the immune reorganization of tumor cells and reboot the immune pathways inhibited by tumors rather than generate tumor immunity. Besides, vaccination is another immunotherapy applicated in cancer, where some tumor-specific antigens like peptides were made into vaccines to trigger the target cytotoxicity. In general, this burgeoning treatment has made a significant improvement in prognosis. Therefore, screening potential patients before treatment is necessary to balance costs and benefits, which means the biomarkers for predicting response are needed.

## 2 Tumor microenvironment

### 2.1 Hypoxia, acidization, and immunosuppression

The tumor microenvironment (TME) refers to the environment around the tumor cells. It plays an essential role in tumorigenesis, which is complicated progress involving multiple interactions between the tumor cell and TME. The cancer cells could secret various cytokines and chemokines to shape TME, leading to the rebuilding of surrounding cells and stroma. For example, TME tended to be acidic with a pH of 6.5-6.9, while the cancer cells and normal tissue presented as basic or neutral. The accumulation of lactic acid due to the Warburg effect was the primary cause of this difference (12). Acidic TME is strongly associated with immune suppression, where immunosuppressive Treg cells could metabolize lactate and thrive in limited glucose concentrations (13). Knockdown of the lactate transporter MCT1 in Treg cells has shown that lactate uptake is dispensable for peripheral normal tissue function, but knockdown in TME can inhibit tumor growth and increase responsiveness to immunotherapy (13). On the other hand, high lactate concentration can trigger apoptosis both *in vivo* and *in vitro* and reduce the infiltration of NK cells, and CTL in TME (14). Lactate receptor GPR81 can be expressed on immune cells and tumor cells, which can promote the secretion of PD-L1 by tumor cells and then affect TME (15).

In addition, the immune-related gene expression could also rebuild the metabolism module of TME. As the PD-L1 signal was recognized, the Akt-mTOR signal pathway in T cells was suppressed, and the translation of glycolysis genes decreased, leading to an inhibition of the glycolytic capacity of T cells (16). Therefore, even with the reorganization of tumor-specific antigens, T cells lacked enough energy to produce a cytolytic effect and cannot kill the tumor cells. Although glycolysis was also inhibited in immunosuppressive Treg cells and M2 macrophages, these cells preferred fatty acids over sugars as energy sources (17). Therefore, immunosuppressed TME was formed by the large consumption of glucose and Warburg effects.

Hypoxia-induced factor (HIF) expression is involved in various tumorigenesis processes. Hypoxia and acidification of TME are both associated with adverse events and survival after chemoradiotherapy (18), and animal models have shown that hypoxia is also an essential factor for immune checkpoint inhibitor resistance (19). According to GEO database analysis, hypoxia and glycolysis are the main risk factors of OS in HNSCC (20). Multiple studies indicated that hypoxia is one of the drivers of immune suppression (18, 21), which can suppress anti-tumor immunity and assist tumors in evading immune surveillance.

Current research showed three main mechanisms of immune escape in hypoxia: 1. The abnormal signal transduction of NO is induced to inhibit the tumor-killing mediated by NK cells and cytotoxic T cells; 2. Regulate the activity of DC, NK, and T cells by inducing adenosine expression; 3. Promote T cell regulation by TAM and other factors in the microenvironment (18). HIF-1 and CAIX are both transcription factors stably expressed in hypoxia TME. They are involved in the genetic transcription related to metabolism, tumor angiogenesis, invasion, and survival. Carbonic anhydrase IX (CAIX) accounted for acidizing TME *via* carbonic acid (22), while HIF promoted the expression of the macrophage cytokines CCL5 and CCL12, which recruited macrophages in TME and polarized them into M2 immunosuppressive macrophages (23). At the same time, hypoxia stimulated tumor cells to secrete IL-10 and TGF-β, leading to the activation and proliferation of Treg cells (24). The activation of these cells built and characterized the immune TME.

Several methods can quantify hypoxia levels, such as oxygen electrodes inserted directly into the tumor, staining with endogenous or exogenous markers, and TH imaging (25). Although the oxygen electrode can directly measure TME oxygen, its sensitivity is limited due to its large size and might also change the oxygen distribution. Endogenous potential hypoxia biomarkers include HIF, erythropoietin EPO, CAIX, glucose transporter GLUT-1/3, etc. Fluoro18 misonidazole positron emission tomography ([18F]FMISO PET) is a common tool used as a non-invasive imaging technique to assess tumor hypoxia (26).

In addition, remission of local tumor hypoxia during early treatment is closely related to high lymphocytic infiltration, in which patients have better local tumor control than patients with low levels (27). This suggests that the immune efficacy may be improved if hypoxia-related gene expression can be alleviated during immunotherapy.

### 2.2 HPV

Different risk factors give tumors intrinsic heterogeneity, especially HPV infection in oropharyngeal cancer. HPV-negative HNSCC tended to have suppressor gene p53 mutation, decreased p16 expression, and amplification of Rb, with chromosome 9p deficiency and 7p duplication, which enhance EGFR expression. In contrast, HPV-positive HNSCC has PI3KCA mutation and chromosome 3q duplication (28). However, the overall mutational burden of HPV-positive and HPV-negative HNSCC was similar, with somatic exonic mutations average of 14.4 and 15.2 respectively (29).

A transcriptional analysis based on the Gene Expression Omnibus (GEO) indicated that HPV-positive HNSCC had increased Treg/CD8 and better OS than HPV-negative (30). HPV viral antigens activated innate immune response and enhanced T and B cell-adaptive immune responses (31). Moreover, HPV could increase the infiltration of T follicular helper cells, γδ T cells, and eosinophils while inhibiting monocytes and neutrophils in TME (32). Both T follicular helper cells and monocytes could secrete multiple cytokines to regulate B cells and develop drug resistance, leading to enhanced tumor immune response (33, 34), and eosinophil was related to improved prognosis (28). HPV-positive patients have a better response of chemotherapy and radiotherapy and fewer coexisting conditions compared with HPV-negative patients (35). Besides, genetic mutations caused by tobacco may impair the treatment response of HPV-positive tumors (36). Although HPV infection plays a role in TME, there was no evidence showing the relationship between HPV status and immunotherapy, especially in PD-L1 inhibitor (37).

### 2.3 Immune tumor microenvironment

Pan-cancer analysis indicated that the immune reaction in the majority of HNSCC is remarkable, and HNSCC has the highest levels of Treg/CD8+ T cell ratio and CD56dim NK cell infiltration (38). The immune tumor environment is an essential factor in immunotherapy, and the entire tumor immune includes seven steps: 1. Release of tumor antigen; 2. Tumor antigen presentation; 3. T cell initiation and activation; 4. T cell migration; 5. Infiltration of T cells into tumor tissue; 6. Tumor recognition by T cells; 7. Cleaning tumor cells (39).

There are serial TME classifications based on academic disciplines such as histology and bioinformation analysis (40, 41). But all those classifications have similar clinical outcome patterns, indicating the immune oncology’s internal relationship. According to the inflammation stage of HNSCC, TME could be divided into three subtypes: inflamed, immune-excluded, and immune-desert (40), which are associated with tumor and mesenchyme immune infiltration, mesenchyme inflammation, and non-inflammation respectively. The promoters of T-cell inflamed tumor immunologic escape include IFN-γ-mediated overexpression of PD-L1 and IDO and FoxP3+ Treg cells. Although the immune function was suppressed in this kind of subgroup, restarting it is feasible, where ICI tried to terminate this inhibition for tumor destruction (42). This means the immunotherapy might only work on immune-infiltrated patients (43). On the other hand, non-inflamed cancer has enhanced WNT-catenin signal pathway and overexpression of BATF3, DCs, and Myc, leading to immune rejection status (44).

The immune TME classification based on the TCGA database has similar outcomes in several analyses (41, 45). *Via* a non-negative matrix factorization algorithm, the immune TME in HNSCC could be divided into non-immune and immune. The immune subgroup has enriched inflammatory response, activated IFN-γ signal, and cytolytic activity. Moreover, the immune group could be divided into active immune and exhausted immune. Although there was no statistical difference in immune enrichment score and IFN-γ signal between those two subgroups, the active immune class had abundant tumor-infiltrating lymphocytes (TILs), M1 macrophage infiltration, enhanced cytotoxicity, HPV infection, and better prognosis, while exhausted immune class was characterized by matrix activation, M2 macrophage infiltration, enhanced WNT signal pathway, and poor prognosis. As confirmed by the melanoma immunotherapy study cohort, the active immune type may suggest potential immunotherapy benefits (45).

In conclusion, although different studies performed different analyses of HNSCC, they all achieved similar results in identifying immunoreactive, immunosuppressed, and non-immune subtypes. However, these genotypes lack analysis of immunotherapy samples for HNSCC and should be further validated to guide immunotherapy.

## 3 The biomarkers of the immune microenvironment

The oncogenesis process involves multiple gene expression mutations. Only one single gene mutation does not cause tumorigenesis directly, which needs more mutations to escape from host defense systems like DNA repair and immune check. In other words, multiple molecules were expressed in the immune microenvironment, and their different interaction patterns rebuild the drug sensibility and resistance (**Table 1**). Unfortunately, a significant proportion of them has a double-edged sword effect on oncogenesis, where they promote this progress in some cancers while suppressing it in others.

**Table 1:** Biomarkers of immune TME in HNSCC and their advantages and disadvantages.

Biomarkers	Method	Expression	Mechanism	Mode of action	Advantages	Disadvantages
PD-L1/PDCD1	CPS/TPS(IHC)	Increased	Suppressed T cell; Escaped immune killing	Exosome	Mature target therapy with clinical predictive biomarkers	The prediction efficiency is not enough, and supplemented biomarkers are needed
				Ligand-receptor		
IFN-γ	Sequencing	Increased	Upstream of PD1	Ligand-receptor	Gene signatures have 95% of negative predictive value	No differentially expression found in baseline samples
					Potential biomarker to exclude immunotherapy	
CXC family	Sequencing	Increased in ICI response	Introduced lymphocyte infiltration into the lesion and inhibited tumor growth	Ligand-receptor	Potential predictive biomarker by pan-cancer analysis	Need verification in untreated HNSCC
TILs	Microarray	Increased in patients with longer DFS, OS, and better locoregional control	Tumor cytolysis and maintained immune surveillance	Cell-to-Cell	Predictive model of TILs and PD-L1	Need verification in untreated HNSCC
	RNA sequencing					
CAFs	Sequencing	Dependent on subgroup	HNCAF-1 Immunosuppression	Cell-to-Cell	HNCAF-0/3 predict PD-1 therapy response	Need verification in larger number of untreated HNSCC
			HNCAF-0/3 stimulated Trem and cytotoxic T cells	Ligand-receptor	Immunosuppressive HNCAF-1 specific in HNSCC	
				Exosome		

### 3.1 Programmed death-ligand 1

The most widely applicated molecule of the immune microenvironment is programmed death ligand 1 (PD-L1), a cytokine secreted from tumor cells. Physiologically, PD-L1 would be expressed on the somatic cellular membrane to suppress autoimmune and avoid overactivation of the immune system. PD-1 acts like a break for the immune system, inhibiting T cell apoptosis rather than tumor-specific antigen recognition. Similarly, the tumor cells could also inhibit immune-induced apoptosis *via* cell-to-cell contact with PD-1. Hence, the host immune system could not destroy the tumor.

Moreover, PD-L1 could rebuild the entire metabolic pattern of the tumor microenvironment, which is an expanded understanding of tumor glycometabolism among the Warburg effect. After recognition of PD-L1, Akt/mTOR pathway in the T cells was inhibited, leading to decreased glycolytic capacity (16). In this situation, the enzymes for glycolysis were less translated, and T cells were lack of enough energy to produce a solid cytolytic immune response even if tumor-specific antigen was recognized. Besides, T-reg cells and M2 macrophages, which could suppress the immune reaction, prefer to take advantage of fatty acid as an energy source rather than glucose (17). Therefore, since the whole tumor microenvironment was in a low glucose concentration, the antigen-presenting and cytotoxicity of the immune system were suppressed while the tumor cells kept proliferating.

Clinically, the use of PD-1 inhibitors is mainly affected by the expression level of PD-1/PD-L1, TMB, and tumor microsatellite instability (MSI). However, the 2018 expert consensus does not recommend testing for MSI before treatment for HNSCC (46). As one of the immunotherapy targets, PD-L1 was successfully applied for pre-treatment screening, whose expression level could be detected by immunohistochemistry (IHC). Tumor proportion score (TPS) and combined positive score (CPS) were two immune indicators to evaluate the potential effects of pembrolizumab. The patients with a higher value of CPS tended to have better prognoses, like more prolonged overall survival (OS), and CPS had a positive correlation with OS in HNSCC (7). TPS was defined as the ratio of partially or entirely stained tumor cells to total tumor cells at any staining intensity, while CPS was defined as the ratio of partially or entirely stained tumor cells, lymphocytes, and macrophages to all tumor cells at any staining intensity. The higher the CPS value, the better the prognosis of patients, and the longer OS, where CPS and OS were positively correlated (7).

TPS and CPS are both suitable indicators but not good enough for predicting the potential therapeutic benefits of ICI entirely because PD-1 inhibitors could prolong median overall survival compared with standard of care regardless of CPS (9). Although the FDA has approved the use of PD-1 inhibitors in patients with CPS≥1, this does not mean that patients with CPS < 1 cannot benefit from PD-1 inhibitors. Patients did not fare worse without pre-treatment screening (median OS HR=0.8 95%CI 0.65 to 0.98) (7). This indicates that the predictive role of CPS as a screening indicator for treatment needs to be improved, and new indicators are urgently needed to predict the efficacy of immunotherapy.

### 3.2 Interferon-γ

Interferon-γ is the only member of the type II class of interferon, which is also the core molecule of the PD-L1 upstream molecules and stimulates PD-L1 expression (47). IFN-γ has been shown to be significantly related to the application of PD-L1 inhibiting therapy and immune TME. IFN-γ plays a critical driver role and could predict the clinical response to PD-1 treatment (48). Current studies of HNSCC immunotherapy showed IFN-γ-related gene difference was the mainstream change (11, 48), while TME classification did not show a significant difference in IFN-γ expression between the active immune class and exhausted immune class (45). Based on RNA from the baseline tumor samples of pembrolizumab-treated patients, eighteen IFN-γ-related genes were identified. Those genes are related to antigen-presenting, chemokine expression, cytotoxic activity, and adaptive immunity (48). KEYNOTE-012 trial studying HNSCC applied six-gene interferon-γ signature from those eighteen genes, including IDO1, CXCL10, CXCL9, HLA-DRA, STAT1, and IFN-γ (10). It is shown that this signature is statistically related to Best Overall Response (BOR) and PFS, and since its negative predictive value is up to 95%, it is a potential biomarker to exclude immunotherapy. Meanwhile, interferon-γ showed its independent predicting potential in the metastatic castration-resistant prostate cancer by prostate-specific antigen response measurement by IFN-γ ELISPOT (49, 50). However, there was no differentially IFN-γ expression found in immune TME of HNSCC prior to treatment (11). This indicates that the immunotherapy-related molecules discovered so far are only the tip of the iceberg, and more potential molecules need to be explored.

### 3.3 IFN-γ related CXC family

CXC chemokine family is one of four cytokine-like molecules, which takes participate in multiple oncogenesis progress, such as tumor angiogenesis, immune cell infiltration, and leukocyte migration. Almost all the subgroups of CXCL increased in HNSCC based on seven datasets, but they have different expression levels and variable functions, resulting in different consequences. For example, CXCL1, 2, 4, 6, 7, 8 were related to short OS, while the patients with CXCL 9,10, 13, 14, 17 tended to have longer OS (51). CXCL9 and CXCL10 are both reported to be significantly expressed in HNSCC, especially in TME and immunotherapy (10, 11). They seemed to be the positive factors for immunotherapy response group, which means patients with higher expression got more therapeutic benefits than who did not have. Besides, the cluster analysis identified CXCL9, 10, 11, and 13 as one cluster (52), all of which are the ligands of CXCR3 receptor and showed considerable predicting effects with AUROC greater than 0.8. Those molecules suggest an immune-hot TME (53), and CXCR3 was mainly expressed on activated CD8 T cells and NK cells (54). But this axis was relevant with proliferation and metastasis in some kinds of cancers like colon cancer and breast cancer (55).

Moreover, CXCL9, -10, -11/CXCR3 axis played an essential role in anti-PD-1 treatment, where anti-PD-1failed to inhibit the tumor growth with CXCR3 knockdown (54). The pan-cancer study showed that CXCL9 as the ICI predictor was better than CD 8 effector and the T cell inflamed signatures (56). There have been many successful applications of CXCL-related inhibitors for suppressing tumor growth and metastasis except HNSCC (55). So, the combined use of PD-1 and CXCL-related inhibitors might be a potential treatment option. However, there was no study that compared the prediction effects of CXCL and IFN-γ and figured out whether CXCL could predict OS independently or just another manifestation of IFN-γ.

### 3.4 Tumor-infiltrating lymphocytes

Tumor-infiltrating lymphocytes (TILs) could be evaluated by microarray or RNA sequencing through deconvolution like CIBERSOR and associated with better prognosis (57, 58). For example, HNSCC patients with high level of TILs had longer DFS, OS, and better locoregional control (59, 60). CD8+ TILs, as potential targets of PD-L1, played an essential role in directly killing tumor cells and maintaining immune surveillance and associated with prolonged OS (61). This lymphocyte subgroup could reflect the TILs, where TILs was moderately correlated with PD1 and CD8a mRNA levels (62). Besides, high expression of CD8+ TILs and CTLA-4 enhanced the response to PD-1 monotherapy and PFS (63). A tumor model based on TILs and PD-L1 was established to predict the immunotherapy response. Double positive expression was most likely to respond to PD-1/PD-L1 inhibitor therapy, while double negative expression showed resistance of PD-1/PD-L1 inhibitor (64). Nevertheless, the number of PD-1 inhibitor application in untreated HNSCC was limited, and those research findings need further verification.

### 3.5 Cancer-associated fibroblasts

Cancer-associated fibroblasts (CAFs) are the most prominent cell type in the TME of many cancers, and they cannot undergo apoptosis due to persisting activation (65). Resent research suggested that CAFs were associated with resistance of chemotherapy and ICI (66–68). Unlike other biomarkers above, the number of CAF subtypes varied in different cancers. For example, there were four CAF subtypes identified *via* the expression of six fibroblast markers, while there were three CAF subtypes in pancreatic cancer according to cytokine and surface marker expression. Most of them were associated with immunosuppression and immunotherapy resistance (66). As for HNSCC, one study, based on samples from baseline and nivolumab treatment, recognized fourteen gene expression clusters, classifying four CAF subtypes named HNCAF-0 to HNCAF-4 (69). After immunotherapy, HNCAF-0 and HNCAF-3 increased while HNCAF-1 and HNCAF-2 decreased (69). Then, VIPER protein activity analysis identified subpopulations associated with response and resistance to ICI. HNCAF-0/3 could stimulate tissue-resident memory T cells (Trem) and cytotoxic T cells *via* cell-to-cell contact, leading to the secretion of perforin, granzyme B, and IFN-gamma. That may explain why HNCAF-0/3 could predict the response of PD-1 therapy and enhance the intrinsic immune. On the other hand, HNCAF-1, which was more specific in HNSCC, could induce apoptosis of T cells, causing immunosuppressive TME (69).

Besides, CAFs were also able to secrete exosomes into the cancer cells, whose cargoes included multidrug-resistant associated proteins, miRNAs, and lncRNAs. Exosomal miR-196a derived from CAFs showed the ability to promote G1/S cell cycle change and suppress apoptosis by inhibiting CDKN1B and ING5 *in vitro* tests (70). Meanwhile, the researcher also found that the plasma exosomal miR-196a, mainly originating from cancer, was corresponded with OS and drug resistance. Exactly, miR-196a from plasma in the drug-resistant group was higher than sensitive group, whose prediction effect was considerable (AUROC=0.828) (70).

## 4 The signal pathways involved in the immune microenvironment

### 4.1 JAK-STAT pathway

Janus kinase-signal transducer and activator of transcription (JAK-STAT) signal pathway takes part in almost all immune regulatory processes, especially in the identification of tumor cells and related immune escape. The role of this pathway in tumors depends on the dominant expression of the STAT subtype, where different STAT subtypes produce different effects. For example, STAT1 and STAT2 were related with innate and adaptive antitumor immunity and promotion of tumor immunogenicity, while STAT3 and STAT5 were related with cancer cell survival, immunosuppression, and sustained inflammation in TME, leading to immune signal loss and M2 macrophage polarization (71). STAT3 and STAT5 were activated in HNSCC and promoted immune system evasion (72). However, the pan-cancer analysis did not show the predictive effects of JAK in PD-L1 treatment, while the failure of single biomarker prediction did not exclude its effect in specific cancer (56). Therefore, relevant activation molecules should be differentiated and stratified in relevant patients in order to obtain a clinically meaningful effect.

### 4.2 HGF/Met signal pathway

The hepatocyte growth factor/mesenchymal-epithelial transition factor (HGF/Met) signal pathway has been wildly studied in TME and immune modulation. This pathway could introduce M1 macrophages into M2 macrophages, which is a promoter of tumorigenesis (73). Besides, HGF impressed the antigen-presenting function of dendritic cells (DC) and proliferation of CD25+Foxp3+ Treg cells. Sun et al. established a dual inhibitor of MET and PD-1 which had strong anti-proliferative and anti-metastatic effects (74). However, this signal pathway and its related immunotherapy need further study especially in untreated HNSCC.

## 5 Potential predictive biomarkers of immunotherapy in microenvironment

As mentioned above, there are multiple biomarkers involved in the immune TME, and one marker could vary in different cancers. Tumor heterogeneity refers to multiple neoplastic characteristics like phenotype and gene expression differences even in the same kind of tumor. This diversity may account for why there are variable ICI therapeutic effects and the reason why predicting biomarkers are not widely applicable. Besides, the research based on the tumor genome cannot reflect the gene expression and clinical parameters like OS directly. For instance, CheckMate-141 showed no statistical significance between PD-L1 expression and clinical outcomes in the platinum-refractory R/M HNSCC (8). It might be explained by the hypothesis that the genome expression had changed after chemotherapy and the tumor cells survived from chemotherapy gain intrinsic ICI resistance. Therefore, the exploration of potential response groups to immunotherapy has yet to be carried out.

In this review, we screened molecular markers with potential predictive significance (**Table 2**). The pan-cancer study indicated that tumor mutational burden (TMB) had the most powerful predictive effects on ICI treatment, especially clonal TMB (56). Tumor immune assessment focus on immune TME and tried to figure out a predicting signature, which could be a supplement to TMB. However, it evaluated part of the immune mechanism and lacked enough HNSCC immunotherapy for efficiency validation. Those biomarkers need clinical validation in tumor samples. Exosomes carried multiple cytokines and regulating RNA. Meanwhile, it could be extracted from peripheral blood for the test. Other peripheral blood-related indicators had shown predicting power in other tumor treatments. Both of noninvasive detections might help in early treatment. Although CTLA-4 is not effective biomarker for HNSCC, the combination of CTLA-4 inhibitors and other agents like PD-L1 inhibitors could increase curative effects. Besides, methylthioadenosine phosphorylase was discovered in HNSCC immunotherapy. Both of two biomarkers need further verification, especially in untreated HNSCC.

**Table 2:** The potential biomarkers in HNSCC immune TME and their advantages and disadvantages.

Potential Biomarker		Method	Functions	Advantages	Disadvantages
TMB		Sequencing	Reflect neoantigen level	Clonal TME was the most powerful predictor of ICI response by pan-cancer analysis	Need verification in untreated HNSCC
IRGPI	SFRP4/CPXM1/COL5A1	Sequencing	Positive with active immune	IRGPI-high group tended to benefit more from ICI therapy	Predicting method TIDE was trained by R/M HNSCC
Exosome	TSP1	Sequencing	Polarization of M1	Responsible for cancer stage and lymph node metastasis	Relationship between exosomes and distant metastasis is controversial
	PD-L1		Immune checkpoint inhibitor		
	miR-196a		Corresponded with OS and drug resistance	Considerable prediction effect	
Peripheral Blood	NLR	Blood Test	Reflect the degree of specific immunity	Increased NLR with shorter OS	Its role in HNSCC immunotherapy needs verification
	Eosinophil		A role of immune TME	Potential for predicting the tumor recurrence	
CTLA-4		Sequencing	Inhibit APC and T cell activation	CTLA-4 inhibitor treatment enhanced immunotherapy	Need verification in HNSCC
MTAP		Sequencing	Break down methylthioadenosine and expression of Toll-like receptor and JAK-STAT signal pathway	predict the low response rate of immune therapy	Limited samples and Need verification in untreated HNSCC

### 5.1 Tumor mutational burden

TMB, a tool to quantify the tumor heterogeneity, is defined as the number of non-inherited mutations per million bases of investigated genomic sequence and detected by “gold standard” Whole-Exon Sequencing (WES). It was regarded as a potential predictive biomarker of ICI therapy response in prospective clinical trials of HNSCC (75). TMB was positive related with OS and could predict the objective response rate in PD-1 inhibition therapy (75). A study based on TCGA database identified around 50-100 mutated genes as candidate cancer driver genes in HNSCC (76), many of which occurred at low frequency and their functions reminded unclearly. Besides, a portion of TMB could generate neoantigen during tumorigenesis, a kind of antigen entirely not present in the human genome. The more TMB tumor has, the more chance of neoantigen forms. The neoantigen could trigger T cell antigen recognition and activation (77), explaining the correlation between TMB and the immune TME (75). A study based on non-small cell lung cancer (NSCLC) and melanoma indicated that the high level of neoantigen clonality leaded to the inflamed TME and strengthened the immunotherapy effects, which means the multiple tumor subclones could increase the possibility of host immune invalidation (78).

TMB has shown its predictive power in immunotherapy, especially ICI. Clonal TMB, defined as the number of nonsynonymous mutations estimated to be present in cancer cell, was the most powerful predictor of ICI response by pan-cancer analysis (56). However, this pan-cancer analysis and most of current clinical trials involved R/M HNSCC rather than untreated HNSCC, where the tumor’s genetic expression had changed and tumor gained resistances to drugs. Hence, the predictive effect of TMB in HNSCC should be verified in the further study.

### 5.2 Tumor immune assessment

Bioinformatics analysis is a wildly applied tool targeting the whole cancer genome to figure out the potential expressed biomarkers. Multiple studies tried to find the relationship between clinical outcomes or TMB with certain biomolecules. An immune-related genetic prognostic index (IRGPI) related to TMB came up as a biomarker to predict the benefit of immunotherapy in HNSCC (79). Three immune-related genes, SFRP4, CPXM1, and COL5A1, were figured out as IRGPI in HNSCC based on TCGA database. IRGPI-high group presented more proportion of active immune, characterized as CD8 cell, naïve CD4 cell, activated memory CD4 cell, resting NK, and M1 macrophage, while IRGPI-low group had exhausted immune with naïve B cell, resting memory CD4 cell, and M2 macrophage (79). Meanwhile, Yue et al. also analyzed the genome signature, where IRGPI-high group had more MHC-I and genome repair expression while IRGPI-low group had more genes related immunosuppress and metastasis. Moreover, IRGPI-high group had better prognosis than IRGPI-low group. Tumor immune dysfunction and exclusion (TIDE) score, a new computational method, was used to evaluate the immune escape of tumor. It assessed two mechanisms involved in immune TME: the T cell dysfunction level in cytotoxic T lymphocyte (CTL) high tumors, and T cell exclusion in CTL low tumors (80). IRGPI-high group tended to have lower TIDE score and benefit more from ICI therapy. However, PD-L1 therapeutic validation set applied in this research was urothelium carcinoma rather than HNSCC and TIDE score focus on CTL rather than the whole immune TME, so the predicting effect was limited since other potential molecules was not taken into consideration.

### 5.3 Exosome

Exosomes are one of the three subgroups of extracellular vesicles produced by mammalian cell, while the other two subgroups are microvesicles and apoptotic vesicles. During the tumorigenesis, multiple tumor cells could regulate the gene expression or signal pathways activation in other cells *via* exosomes, where different cargos like miRNA were loaded (81). For example, oral squamous carcinoma cell could induce polarization of macrophages into M1-like phenotype *via* transferring TSP1. While M1 normally suppress tumor cells by inducing inflammation, the exosome activated M1 macrophages could facilitate the migration of tumor cells (82).

The recipient cells uptake exosomes by endocytosis, receptor-ligand interaction, or cell membrane fusion. Among them, the function of receptor-ligand interaction is more than identifying the cargo in exosome. This interaction could activate the signal pathway and gene expression. PD-L1 in exosome was expressed on the membrane of exosomes rather than in the cavity (83). Hence, exosomes were related to prognosis and used for anti-PDL1 therapeutic effect prediction (84). An experiment *in vitro* showed PD-L1 was one of the main immune molecules created from HNSCC in exosomes, and it was PD-L1 exosome in plasma rather than soluble PD-L1 that was connected with cancer stage and lymph node metastasis (85). However, there was a debate of the relationship between exosomes and distant metastasis in clinical. It was proved that not only PD-L1, but also PD-1 receptor could influence 3-year survival, where patients without receptor expression had better prognosis (86).

### 5.4 Peripheral blood related indicators

The systemic tumor immune environment (STIE) is mainly composed of immunomodulatory molecules and immune cells, controlled by circulating blood and lymphatic vessels, and can communicate with the tumor at the primary site and the immune organs of the host. As mentioned before, hypoxia and acid acclamation in TME are like twins, whose are both born from leaky vascular system in TME. The cancer cells could overexpress multiple angiogenic growth factors (TAF), like VEGF. The vessels grow so fast that they do not have enough to be mature or vascularized totally, leading to formation of leaky blood vessels. Therefore, the large molecules like cytokines from cancer cells are easy to spread distantly and this characteristic might help the delivery of drugs and tumor-related antigen presenting, which provides the possibility for peripheral blood to indicate tumor status and predict immune efficacy (11).

Neutrophil-to-lymphocyte ratio (NLR) is a well-studied parameter in peripheral blood which reflects the degree of specific immunity. A meta-analysis including 6479 patients in HNSCC showed that increased NLR was related with shorter OS. In this study, different subgroups of HNSCC, including oral cavity, nasopharynx, hypopharynx, and larynx, were described. It is figured out that the overall HR of OS was 1.78 with 95%CI 1.53-2.07, and the patients with elevated NLR had worse prognosis (87).

Eosinophil as one of the components of peripheral blood was also infiltrating TME, which had shown the potential benefits for predicting the tumor recurrence. During intravesical BCG immunotherapy in non-muscle invasive bladder cancer, increased eosinophil count and percentage before treatment was related with tumor recurrence (88). However, the eosinophil role and predictive effect could change in PD-1 inhibitor immunotherapy of different cancer. In advanced melanoma patients with PD-1 treatment, eosinophil was an independent prognostic factor of OS (89), and it might promote the outcomes of this immunotherapy. This effect might apply to HNSCC, but it needs to be studied and confirmed, especially in immunotherapy.

### 5.5 Cytotoxic T lymphocyte-associated antigen-4

Cytotoxic T lymphocyte-associated antigen-4 (CTLA-4) is widely known immune molecule involved in tumorigenesis. Unlike PD-1, CTLA-4 could inhibit antigen-presenting cells (APC) and the activation of T cells as the competitive antagonist of CD-28, expressed on Tregs. The preclinical trials indicated that adding CTLA-4 inhibitor treatment might enhance the immunotherapy (90, 91), although a few studies reported significant CTLA-4 expression in HNSCC (92). But the CTLA-4 therapy efficiency was still in controversy (93), since most of the current analysis were based on R/M HNSCC. Therefore, further study needs to discover the application of CTLA-4 in untreated HNSCC.

### 5.6 Methylthioadenosine phosphorylase

Methylthioadenosine phosphorylase (MTAP) could catalyze the breakdown of methylthioadenosine intracellularly and reproduce adenine and methionine, which build DNA and RNA. The encoding gene is located on chromosome 9, where the interferon-alpha gene lies. This co-location may explain how the loss of MTAP cause dysfunction of STAT1 and suppress IFN-mediated gene function (94). In the past decades, MTAP has been identified as a purine metabolic enzyme deficient in multiple cancers (95). During the oncogenesis, MTAP mutation interferes with the nucleic acid metabolism, IFN pathway, and immune TME.

Although MTAP was not correlative with immune directly, it showed a potential relevance with immunotherapy. In a study based on ipilimumab monotherapy in melanoma patients, the loss of the IFN-γ signal pathway, including MTAP alteration, could predict the low response rate of immunotherapy. Those patients with dysfunction of IFN-γ showed worse ORR and prognosis (96). Meanwhile, another study involving twenty-nine patients of R/M HNSCC with afatinib and pembrolizumab treatment showed that MTAP was identified as a predicting candidate gene, and MTAP mutation was mainly related with decreased expression of Toll-like receptor and JAK-STAT signal pathway. Tumor cells with MTAP mutation had fewer CD8+ T cells in TME. Moreover, both this dataset and TCGA database indicated that the patients with MTAP mutation had a worse prognosis (97). However, the number of patients in co-treatment of afatinib and pembrolizumab was limited, considering only twenty-nine pre-treatment samples and nine post-treatment samples, which leads to tumor heterogeneity somehow. For example, there was no patient’s TMB over 10 mutations/Mb, whereas 10.1% of patients in the TCGA database had more than 20 mutations/Mb, as described before. Meanwhile, the gene analysis showed no relationship between MTAP and IFN-γ. It is hard to tell if this heterogeneity comes from the limited patient number or the application of afatinib. In other words, MTAP showed its potential, but further study needs to confirm that.

## 6 Conclusion and further study

All the molecules described above are potential biomarkers as therapeutic targets or predicting response to immunotherapy. Clonal TME was the most potential predictor of ICI treatment and most likely to be validated successfully in HNSCC. IRGPI was a good tool to investigate immune TME and patients with IRGPI-high tended to get more benefits from ICI. Exosome with different cargos was responsible for lymph node metastasis and generated considerable prediction effect. NLR could reflect the specific immunity and was associated to OS. Eosinophil as a role of immune TME could predict the tumor recurrence. CTLA-4 was one of the immunotherapy targets, and its inhibitors could enhance anti-PD-L1 response. Although MTAP did not play a direct role in immune TME, it was discovered in untreated HNSCC and could predict low immunotherapeutic response. However, all studies have their limitations. Firstly, almost all studies were based on R/M HNSCC or other kind of cancer, rather than untreated HNSCC. Secondly, the number of patients involved in immunotherapy was always limited, which increased the latent error. Thirdly, the different standards made it hard to compare other samples from various studies.

All of those biomarkers were effective and new biomarker would come up based on initial immunotherapy of HNSCC. Unfortunately, the clinical applications were still finite. Further studies should combine those biomarkers with the clinical samples and data to verify the effectiveness in untreated HNSCC and centralize how to dig out new biomarkers and the patients with a high likelihood of getting more benefits from immunotherapy.

## Author contributions

Writing—original draft: SC. Writing—review and editing: YY, SH, ML, RW, JF. Supervision: JF. All authors contributed to the article and approved the submitted version.

## References

[1] Bray F Ferlay J Soerjomataram I Siegel RL Torre LA Jemal A. Global cancer statistics 2018: GLOBOCAN estimates of incidence and mortality worldwide for 36 cancers in 185 countries. CA Cancer J Clin (2018) 68(6):394–424. doi: 10.3322/caac.21492 30207593

[2] Ferlay J Soerjomataram I Dikshit R Eser S Mathers C Rebelo M. Cancer incidence and mortality worldwide: sources, methods and major patterns in GLOBOCAN 2012. Int J Cancer (2015) 136(5):E359–86. doi: 10.1002/ijc.29210 25220842

[3] Mourad M Jetmore T Jategaonkar AA Moubayed S Moshier E Urken ML. Epidemiological trends of head and neck cancer in the united states: A SEER population study. J Oral Maxillofac Surg (2017) 75(12):2562–72. doi: 10.1016/j.joms.2017.05.008 PMC6053274 28618252

[4] Global Burden of Disease Cancer C Fitzmaurice C Allen C Barber RM Barregard L Bhutta ZA. Global, regional, and national cancer incidence, mortality, years of life lost, years lived with disability, and disability-adjusted life-years for 32 cancer groups, 1990 to 2015: A systematic analysis for the global burden of disease study. JAMA Oncol (2017) 3(4):524–48. doi: 10.1001/jamaoncol.2016.5688 PMC6103527 27918777

[5] Gatta G Botta L Sanchez MJ Anderson LA Pierannunzio D Licitra L. Prognoses and improvement for head and neck cancers diagnosed in Europe in early 2000s: The EUROCARE-5 population-based study. Eur J Cancer (2015) 51(15):2130–43. doi: 10.1016/j.ejca.2015.07.043 26421817

[6] Canning M Guo G Yu M Myint C Groves MW Byrd JK. Heterogeneity of the head and neck squamous cell carcinoma immune landscape and its impact on immunotherapy. Front Cell Dev Biol (2019) 7:52. doi: 10.3389/fcell.2019.00052 31024913 PMC6465325

[7] Burtness B Harrington KJ Greil R Soulières D Tahara M de Castro G. Pembrolizumab alone or with chemotherapy versus cetuximab with chemotherapy for recurrent or metastatic squamous cell carcinoma of the head and neck (KEYNOTE-048): a randomised, open-label, phase 3 study. Lancet (2019) 394(10212):1915–28. doi: 10.1016/S0140-6736(19)32591-7 31679945

[8] Ferris RL Blumenschein G Jr. Fayette J Guigay J Colevas AD Licitra L. Nivolumab vs investigator's choice in recurrent or metastatic squamous cell carcinoma of the head and neck: 2-year long-term survival update of CheckMate 141 with analyses by tumor PD-L1 expression. Oral Oncol (2018) 81:45–51. doi: 10.1016/j.oraloncology.2018.04.008 29884413 PMC6563923

[9] Cohen EEW Soulières D Le Tourneau C Dinis J Licitra L Ahn M-J. Pembrolizumab versus methotrexate, docetaxel, or cetuximab for recurrent or metastatic head-and-neck squamous cell carcinoma (KEYNOTE-040): a randomised, open-label, phase 3 study. Lancet (2019) 393(10167):156–67. doi: 10.1016/S0140-6736(18)31999-8 30509740

[10] Seiwert TY Burtness B Mehra R Weiss J Berger R Eder JP. Safety and clinical activity of pembrolizumab for treatment of recurrent or metastatic squamous cell carcinoma of the head and neck (KEYNOTE-012): an open-label, multicentre, phase 1b trial. Lancet Oncol (2016) 17(7):956–65. doi: 10.1016/S1470-2045(16)30066-3 27247226

[11] Uppaluri R Campbell KM Egloff AM Zolkind P Skidmore ZL Nussenbaum B. Neoadjuvant and adjuvant pembrolizumab in resectable locally advanced, human papillomavirus-unrelated head and neck cancer: A multicenter, phase II trial. Clin Cancer Res (2020) 26(19):5140–52. doi: 10.1158/1078-0432.CCR-20-1695 PMC7547532 32665297

[12] Griffiths JR. Are cancer cells acidic? Br J Cancer (1991) 64(3):425–7. doi: 10.1038/bjc.1991.326 PMC1977628 1911181

[13] Watson MJ Vignali PDA Mullett SJ Overacre-Delgoffe AE Peralta RM Grebinoski S. Metabolic support of tumour-infiltrating regulatory T cells by lactic acid. Nature (2021) 591(7851):645–51. doi: 10.1038/s41586-020-03045-2 PMC7990682 33589820

[14] Brand A Singer K Koehl GE Kolitzus M Schoenhammer G Thiel A. LDHA-associated lactic acid production blunts tumor immunosurveillance by T and NK cells. Cell Metab (2016) 24(5):657–71. doi: 10.1016/j.cmet.2016.08.011 27641098

[15] Feng J Yang H Zhang Y Wei H Zhu Z Zhu B. Tumor cell-derived lactate induces TAZ-dependent upregulation of PD-L1 through GPR81 in human lung cancer cells. Oncogene (2017) 36(42):5829–39. doi: 10.1038/onc.2017.188 28604752

[16] Chang CH Qiu J O'Sullivan D Buck MD Noguchi T Curtis JD. Metabolic competition in the tumor microenvironment is a driver of cancer progression. Cell (2015) 162(6):1229–41. doi: 10.1016/j.cell.2015.08.016 PMC4864363 26321679

[17] Huang SC Everts B Ivanova Y O'Sullivan D Nascimento M Smith AM. Cell-intrinsic lysosomal lipolysis is essential for alternative activation of macrophages. Nat Immunol (2014) 15(9):846–55. doi: 10.1038/ni.2956 PMC4139419 25086775

[18] Lee CT Mace T Repasky EA. Hypoxia-driven immunosuppression: A new reason to use thermal therapy in the treatment of cancer? Int J Hyperthermia (2010) 26(3):232–46. doi: 10.3109/02656731003601745 PMC2909041 20388021

[19] Zandberg DP Menk AV Velez M Normolle D DePeaux K Liu A. Tumor hypoxia is associated with resistance to PD-1 blockade in squamous cell carcinoma of the head and neck. J Immunother Cancer (2021) 9(5):e002088. doi: 10.1136/jitc-2020-002088 33986123 PMC8126285

[20] Liu J Lu J Li W. Transcriptome analysis reveals the prognostic and immune infiltration characteristics of glycolysis and hypoxia in head and neck squamous cell carcinoma. BMC Cancer (2022) 22(1):352. doi: 10.1186/s12885-022-09449-9 35361159 PMC8969218

[21] Duechler M Peczek L Zuk K Zalesna I Jeziorski A Czyz M. The heterogeneous immune microenvironment in breast cancer is affected by hypoxia-related genes. Immunobiology (2014) 219(2):158–65. doi: 10.1016/j.imbio.2013.09.003 24091277

[22] Lee SH Griffiths JR. How and why are cancers acidic? carbonic anhydrase IX and the homeostatic control of tumour extracellular pH. Cancers (Basel) (2020) 12(6):1616. doi: 10.3390/cancers12061616 32570870 PMC7352839

[23] Betts GN Eustace A Patiar S Valentine HR Irlam J Ramachandran A. Prospective technical validation and assessment of intra-tumour heterogeneity of a low density array hypoxia gene profile in head and neck squamous cell carcinoma. Eur J Cancer (2013) 49(1):156–65. doi: 10.1016/j.ejca.2012.07.028 22951015

[24] Deng B Zhu JM Wang Y Liu TT Ding YB Xiao WM. Intratumor hypoxia promotes immune tolerance by inducing regulatory T cells *via* TGF-beta1 in gastric cancer. PloS One (2013) 8(5):e63777. doi: 10.1371/journal.pone.0063777 23723999 PMC3664556

[25] Curtis KK Wong WW Ross HJ. Past approaches and future directions for targeting tumor hypoxia in squamous cell carcinomas of the head and neck. Crit Rev Oncol Hematol (2016) 103:86–98. doi: 10.1016/j.critrevonc.2016.05.005 27247118

[26] Kadrmas DJ Hoffman JM. Methodology for quantitative rapid multi-tracer PET tumor characterizations. Theranostics (2013) 3(10):757–73. doi: 10.7150/thno.5201 PMC3840410 24312149

[27] Nicolay NH Ruhle A Wiedenmann N Niedermann G Mix M Weber WA. Lymphocyte infiltration determines the hypoxia-dependent response to definitive chemoradiation in head-and-Neck cancer: Results from a prospective imaging trial. J Nucl Med (2021) 62(4):471–8. doi: 10.2967/jnumed.120.248633 PMC8049369 32859699

[28] Xu L Jin Y Qin X. Comprehensive analysis of significant genes and immune cell infiltration in HPV-related head and neck squamous cell carcinoma. Int Immunopharmacol (2020) 87:106844. doi: 10.1016/j.intimp.2020.106844 32738592

[29] Seiwert TY Zuo Z Keck MK Khattri A Pedamallu CS Stricker T. Integrative and comparative genomic analysis of HPV-positive and HPV-negative head and neck squamous cell carcinomas. Clin Cancer Res (2015) 21(3):632–41. doi: 10.1158/1078-0432.CCR-13-3310 PMC4305034 25056374

[30] Keck MK Zuo Z Khattri A Stricker TP Brown CD Imanguli M. Integrative analysis of head and neck cancer identifies two biologically distinct HPV and three non-HPV subtypes. Clin Cancer Res (2015) 21(4):870–81. doi: 10.1158/1078-0432.CCR-14-2481 25492084

[31] Kobayashi K Hisamatsu K Suzui N Hara A Tomita H Miyazaki T. A review of HPV-related head and neck cancer. J Clin Med (2018) 7(9):241. doi: 10.3390/ijms21010059 30150513 PMC6162868

[32] Cillo AR Kurten CHL Tabib T Qi Z Onkar S Wang T. Immune landscape of viral- and carcinogen-driven head and neck cancer. Immunity (2020) 52(1):183–99.e9. doi: 10.1016/j.immuni.2019.11.014 31924475 PMC7201194

[33] Hollern DP Xu N Thennavan A Glodowski C Garcia-Recio S Mott KR. B cells and T follicular helper cells mediate response to checkpoint inhibitors in high mutation burden mouse models of breast cancer. Cell (2019) 179(5):1191–206.e21. doi: 10.1016/j.cell.2019.10.028 31730857 PMC6911685

[34] Komarova EY Marchenko LV Zhakhov AV Nikotina AD Aksenov ND Suezov RV. Extracellular Hsp70 reduces the pro-tumor capacity of Monocytes/Macrophages Co-cultivated with cancer cells. Int J Mol Sci (2019) 21(1):59. doi: 10.3390/ijms21010059 31861801 PMC6982218

[35] Chaturvedi AK Engels EA Pfeiffer RM Hernandez BY Xiao W Kim E. Human papillomavirus and rising oropharyngeal cancer incidence in the united states. J Clin Oncol (2011) 29(32):4294–301. doi: 10.1200/JCO.2011.36.4596 PMC3221528 21969503

[36] Ang KK Harris J Wheeler R Weber R Rosenthal DI Nguyen-Tan PF. Human papillomavirus and survival of patients with oropharyngeal cancer. N Engl J Med (2010) 363(1):24–35. doi: 10.1056/NEJMoa0912217 20530316 PMC2943767

[37] Shamseddine AA Burman B Lee NY Zamarin D Riaz N. Tumor immunity and immunotherapy for HPV-related cancers. Cancer Discovery (2021) 11(8):1896–912. doi: 10.1158/2159-8290.CD-20-1760 PMC8338882 33990345

[38] Mandal R Senbabaoglu Y Desrichard A Havel JJ Dalin MG Riaz N. The head and neck cancer immune landscape and its immunotherapeutic implications. JCI Insight (2016) 1(17):e89829. doi: 10.1172/jci.insight.89829 27777979 PMC5070962

[39] Chen DS Mellman I. Oncology meets immunology: the cancer-immunity cycle. Immunity (2013) 39(1):1–10. doi: 10.1016/j.immuni.2013.07.012 23890059

[40] Chen DS Mellman I. Elements of cancer immunity and the cancer-immune set point. Nature (2017) 541(7637):321–30. doi: 10.1038/nature21349 28102259

[41] Li B Cui Y Nambiar DK Sunwoo JB Li R. The immune subtypes and landscape of squamous cell carcinoma. Clin Cancer Res (2019) 25(12):3528–37. doi: 10.1158/1078-0432.CCR-18-4085 PMC6571041 30833271

[42] Gavrielatou N Doumas S Economopoulou P Foukas PG Psyrri A. Biomarkers for immunotherapy response in head and neck cancer. Cancer Treat Rev (2020) 84:101977. doi: 10.1016/j.ctrv.2020.101977 32018128

[43] Gajewski TF. The next hurdle in cancer immunotherapy: Overcoming the non-T-Cell-Inflamed tumor microenvironment. Semin Oncol (2015) 42(4):663–71. doi: 10.1053/j.seminoncol.2015.05.011 PMC4555998 26320069

[44] Spranger S Gajewski TF. Impact of oncogenic pathways on evasion of antitumour immune responses. Nat Rev Cancer (2018) 18(3):139–47. doi: 10.1038/nrc.2017.117 PMC6685071 29326431

[45] Chen YP Wang YQ Lv JW Li YQ Chua MLK Le QT. Identification and validation of novel microenvironment-based immune molecular subgroups of head and neck squamous cell carcinoma: implications for immunotherapy. Ann Oncol (2019) 30(1):68–75. doi: 10.1093/annonc/mdy470 30407504

[46] Cohen EEW Bell RB Bifulco CB Burtness B Gillison ML Harrington KJ. The society for immunotherapy of cancer consensus statement on immunotherapy for the treatment of squamous cell carcinoma of the head and neck (HNSCC). J Immunother Cancer (2019) 7(1):184. doi: 10.1186/s40425-019-0662-5 31307547 PMC6632213

[47] Abiko K Matsumura N Hamanishi J Horikawa N Murakami R Yamaguchi K. IFN-gamma from lymphocytes induces PD-L1 expression and promotes progression of ovarian cancer. Br J Cancer (2015) 112(9):1501–9. doi: 10.1038/bjc.2015.101 PMC4453666 25867264

[48] Ayers M Lunceford J Nebozhyn M Murphy E Loboda A Kaufman DR. IFN-gamma-related mRNA profile predicts clinical response to PD-1 blockade. J Clin Invest (2017) 127(8):2930–40. doi: 10.1172/JCI91190 PMC5531419 28650338

[49] Kongsted P Borch TH Ellebaek E Iversen TZ Andersen R Met O. Dendritic cell vaccination in combination with docetaxel for patients with metastatic castration-resistant prostate cancer: A randomized phase II study. Cytotherapy (2017) 19(4):500–13. doi: 10.1016/j.jcyt.2017.01.007 28215654

[50] Nixon AB Schalper KA Jacobs I Potluri S Wang IM Fleener C. Peripheral immune-based biomarkers in cancer immunotherapy: Can we realize their predictive potential? J Immunother Cancer (2019) 7(1):325. doi: 10.1186/s40425-019-0799-2 31775882 PMC6880594

[51] Jing F WJ Zhou L Ning Y Xu S Zhu Y. Bioinformatics analysis of the role of CXC ligands in the microenvironment of head and neck tumor. Aging (Albany NY) (2021) 13(13):17789–817. doi: 10.18632/aging.203269 PMC8312447 34247149

[52] Marcovecchio PM Thomas G Salek-Ardakani S. CXCL9-expressing tumor-associated macrophages: new players in the fight against cancer. J Immunother Cancer (2021) 9(2):e002045. doi: 10.1136/jitc-2020-002045 33637602 PMC7919587

[53] Wu X Gu Z Chen Y Chen B Chen W Weng L. Application of PD-1 blockade in cancer immunotherapy. Comput Struct Biotechnol J (2019) 17:661–74. doi: 10.1016/j.csbj.2019.03.006 PMC6558092 31205619

[54] Tokunaga R Zhang W Naseem M Puccini A Berger MD Soni S. CXCL9, CXCL10, CXCL11/CXCR3 axis for immune activation - a target for novel cancer therapy. Cancer Treat Rev (2018) 63:40–7. doi: 10.1016/j.ctrv.2017.11.007 PMC5801162 29207310

[55] Cambien B Karimdjee BF Richard-Fiardo P Bziouech H Barthel R Millet MA. Organ-specific inhibition of metastatic colon carcinoma by CXCR3 antagonism. Br J Cancer (2009) 100(11):1755–64. doi: 10.1038/sj.bjc.6605078 PMC2695685 19436305

[56] Litchfield K Reading JL Puttick C Thakkar K Abbosh C Bentham R. Meta-analysis of tumor- and T cell-intrinsic mechanisms of sensitization to checkpoint inhibition. Cell (2021) 184(3):596–614.e14. doi: 10.1016/j.cell.2021.01.002 33508232 PMC7933824

[57] Gooden MJ de Bock GH Leffers N Daemen T Nijman HW. The prognostic influence of tumour-infiltrating lymphocytes in cancer: A systematic review with meta-analysis. Br J Cancer (2011) 105(1):93–103. doi: 10.1038/bjc.2011.189 21629244 PMC3137407

[58] Newman AM Liu CL Green MR Gentles AJ Feng W Xu Y. Robust enumeration of cell subsets from tissue expression profiles. Nat Methods (2015) 12(5):453–7. doi: 10.1038/nmeth.3337 PMC4739640 25822800

[59] de Ruiter EJ Ooft ML Devriese LA Willems SM. The prognostic role of tumor infiltrating T-lymphocytes in squamous cell carcinoma of the head and neck: A systematic review and meta-analysis. Oncoimmunology (2017) 6(11):e1356148. doi: 10.1080/2162402X.2017.1356148 29147608 PMC5674970

[60] Xu Q Wang C Yuan X Feng Z Han Z. Prognostic value of tumor-infiltrating lymphocytes for patients with head and neck squamous cell carcinoma. Transl Oncol (2017) 10(1):10–6. doi: 10.1016/j.tranon.2016.10.005 PMC5123038 27888708

[61] Solomon B Young RJ Bressel M Urban D Hendry S Thai A. Prognostic significance of PD-L1(+) and CD8(+) immune cells in HPV(+) oropharyngeal squamous cell carcinoma. Cancer Immunol Res (2018) 6(3):295–304. doi: 10.1158/2326-6066.CIR-17-0299 29378694

[62] Prat A Navarro A Pare L Reguart N Galvan P Pascual T. Immune-related gene expression profiling after PD-1 blockade in non-small cell lung carcinoma, head and neck squamous cell carcinoma, and melanoma. Cancer Res (2017) 77(13):3540–50. doi: 10.1158/0008-5472.CAN-16-3556 28487385

[63] Daud AI Loo K Pauli ML Sanchez-Rodriguez R Sandoval PM Taravati K. Tumor immune profiling predicts response to anti-PD-1 therapy in human melanoma. J Clin Invest (2016) 126(9):3447–52. doi: 10.1172/JCI87324 PMC5004965 27525433

[64] Teng MWL Ngiow SF Ribas A Smyth MJ. Classifying cancers based on T-cell infiltration and PD-L1. Cancer Res (2015) 75(11):2139–45. doi: 10.1158/0008-5472.CAN-15-0255 PMC4452411 25977340

[65] De Veirman K Rao L De Bruyne E Menu E Van Valckenborgh E Van Riet I. Cancer associated fibroblasts and tumor growth: focus on multiple myeloma. Cancers (Basel) (2014) 6(3):1363–81. doi: 10.3390/cancers6031363 PMC4190545 24978438

[66] Elyada E Bolisetty M Laise P Flynn WF Courtois ET Burkhart RA. Cross-species single-cell analysis of pancreatic ductal adenocarcinoma reveals antigen-presenting cancer-associated fibroblasts. Cancer Discovery (2019) 9(8):1102–23. doi: 10.1158/2159-8290.CD-19-0094 PMC6727976 31197017

[67] Ohlund D Handly-Santana A Biffi G Elyada E Almeida AS Ponz-Sarvise M. Distinct populations of inflammatory fibroblasts and myofibroblasts in pancreatic cancer. J Exp Med (2017) 214(3):579–96. doi: 10.1084/jem.20162024 PMC5339682 28232471

[68] Kieffer Y Hocine HR Gentric G Pelon F Bernard C Bourachot B. Single-cell analysis reveals fibroblast clusters linked to immunotherapy resistance in cancer. Cancer Discovery (2020) 10(9):1330–51. doi: 10.1158/2159-8290.CD-19-1384 32434947

[69] Obradovic A Graves DA-O Korrer MA-O Wang YA-O Roy S Naveed AA-O. Immunostimulatory cancer-associated fibroblast subpopulations can predict immunotherapy response in head and neck cancer. Clin Cancer Res (2022) 28(10):94–109. doi: 10.1158/1078-0432.CCR-21-3570 PMC9161438 35262677

[70] Qin X Guo H Wang X Zhu X Yan M Wang X. Exosomal miR-196a derived from cancer-associated fibroblasts confers cisplatin resistance in head and neck cancer through targeting CDKN1B and ING5. Genome Biol (2019) 20(1):12. doi: 10.1186/s13059-018-1604-0 30642385 PMC6332863

[71] Owen KL Brockwell NK Parker BS. JAK-STAT signaling: A double-edged sword of immune regulation and cancer progression. Cancers (Basel) (2019) 11(12):2002. doi: 10.3390/cancers11122002 31842362 PMC6966445

[72] Lai SY Johnson FM. Defining the role of the JAK-STAT pathway in head and neck and thoracic malignancies: Implications for future therapeutic approaches. Drug Resistance Updates (2010) 13(3):67–78. doi: 10.1016/j.drup.2010.04.001 20471303

[73] Rutella S Bonanno G Procoli A Mariotti A de Ritis DG Curti A. Hepatocyte growth factor favors monocyte differentiation into regulatory interleukin (IL)-10++IL-12low/neg accessory cells with dendritic-cell features. Blood (2006) 108(1):218–27. doi: 10.1182/blood-2005-08-3141 16527888

[74] Sun ZJ Wu Y Hou WH Wang YX Yuan QY Wang HJ. A novel bispecific c-MET/PD-1 antibody with therapeutic potential in solid cancer. Oncotarget (2017) 8(17):29067–79. doi: 10.18632/oncotarget.16173 PMC5438713 28404966

[75] Yarchoan M Hopkins A Jaffee EM. Tumor mutational burden and response rate to PD-1 inhibition. N Engl J Med (2017) 377(25):2500–1. doi: 10.1056/NEJMc1713444 PMC6549688 29262275

[76] Leemans CR Snijders PJF Brakenhoff RH. The molecular landscape of head and neck cancer. Nat Rev Cancer (2018) 18(5):269–82. doi: 10.1038/nrc.2018.11 29497144

[77] Schumacher TN SR. Neoantigens in cancer immunotherapy. Science (2015) 348(6230):69–74. doi: 10.1126/science.aaa4971 25838375

[78] McGranahan N Furness AJ Rosenthal R Ramskov S Lyngaa R Saini SK. Clonal neoantigens elicit T cell immunoreactivity and sensitivity to immune checkpoint blockade. Science (2016) 351(6280):1463–9. doi: 10.1126/science.aaf1490 PMC4984254 26940869

[79] Chen Y Li ZY Zhou GQ Sun Y. An immune-related gene prognostic index for head and neck squamous cell carcinoma. Clin Cancer Res (2021) 27(1):330–41. doi: 10.1158/1078-0432.CCR-20-2166 33097495

[80] Jiang P Gu S Pan D Fu J Sahu A Hu X. Signatures of T cell dysfunction and exclusion predict cancer immunotherapy response. Nat Med (2018) 24(10):1550–8. doi: 10.1038/s41591-018-0136-1 PMC6487502 30127393

[81] Zhao H Yang L Baddour J Achreja A Bernard V Moss T. Tumor microenvironment derived exosomes pleiotropically modulate cancer cell metabolism. Elife (2016) 5:e10250. doi: 10.7554/eLife.10250 26920219 PMC4841778

[82] Xiao M Zhang J Chen W Chen W. M1-like tumor-associated macrophages activated by exosome-transferred THBS1 promote malignant migration in oral squamous cell carcinoma. J Exp Clin Cancer Res (2018) 37(1):143. doi: 10.1186/s13046-018-0815-2 29986759 PMC6038304

[83] Poggio M Hu T Pai CC Chu B Belair CD Chang A. Suppression of exosomal PD-L1 induces systemic anti-tumor immunity and memory. Cell (2019) 177(2):414–27.e13. doi: 10.1016/j.cell.2019.02.016 30951669 PMC6499401

[84] Zhang L Yu D. Exosomes in cancer development, metastasis, and immunity. Biochim Biophys Acta Rev Cancer (2019) 1871(2):455–68. doi: 10.1016/j.bbcan.2019.04.004 PMC6542596 31047959

[85] Theodoraki MN Yerneni SS Hoffmann TK Gooding WE Whiteside TL. Clinical significance of PD-L1(+) exosomes in plasma of head and neck cancer patients. Clin Cancer Res (2018) 24(4):896–905. doi: 10.1158/1078-0432.CCR-17-2664 29233903 PMC6126905

[86] Gao Q Liu HT Xu YQ Zhang L Liu YR Ren Q. Serum-derived exosomes promote CD8+ T cells to overexpress PD-1, affecting the prognosis of hypopharyngeal carcinoma. Cancer Cell Int (2021) 21(1):584. doi: 10.1186/s12935-021-02294-z 34717645 PMC8557583

[87] Mascarella MA Mannard E Silva SD Zeitouni A. Neutrophil-to-lymphocyte ratio in head and neck cancer prognosis: A systematic review and meta-analysis. Head Neck (2018) 40(5):1091–100. doi: 10.1002/hed.25075 29356179

[88] Temiz MZ Colakerol A Ulus I Kilic E Paslanmaz F Sahin S. Prediction of non-muscle-invasive bladder cancer recurrence during intravesical BCG immunotherapy by use of peripheral blood eosinophil count and percentage: A preliminary report. Cancer Immunol Immunother (2021) 70(1):245–52. doi: 10.1007/s00262-020-02673-x PMC10991931 32700089

[89] Kartolo A Holstead R Hopman W Baetz T. Prognosticating role of serum eosinophils on immunotherapy efficacy in patients with advanced melanoma. Immunotherapy. (2021) 13(3):217–25. doi: 10.2217/imt-2020-0265 33238773

[90] Seiwert TY Weiss J Baxi SS Ahn M-J Fayette J Gillison ML. A phase 3, randomized, open-label study of first-line durvalumab (MEDI4736) ± tremelimumab versus standard of care (SoC; EXTREME regimen) in recurrent/metastatic (R/M) SCCHN: KESTREL. J Clin Oncol (2016) 34(15_suppl):TPS6101–TPS. 10.1016/j.annonc.2022.12.008 36535565

[91] Siu L Even C Mesía R Daste A Krauss J Saba NF. A randomized, open-label, multicenter, global phase 2 study of durvalumab (D), tremelimumab (T), or d plus T, in patients with PD-L1 Low/Negative recurrent or metastatic head and neck squamous cell carcinoma: CONDOR. Int J Radiat OncologyBiologyPhysics (2018) 100(5).

[92] Chen J Yang J Li H Yang Z Zhang X Li X. Single-cell transcriptomics reveal the intratumoral landscape of infiltrated T-cell subpopulations in oral squamous cell carcinoma. Mol Oncol (2021) 15(4):866–86. doi: 10.1002/1878-0261.12910 PMC8024729 33513276

[93] Ferris RL Haddad R Even C Tahara M Dvorkin M Ciuleanu TE. Durvalumab with or without tremelimumab in patients with recurrent or metastatic head and neck squamous cell carcinoma: EAGLE, a randomized, open-label phase III study. Ann Oncol (2020) 31(7):942–50. doi: 10.1016/j.annonc.2020.04.001 32294530

[94] Mowen KA Tang J Fau - Zhu W Zhu W Fau - Schurter BT Schurter Bt Fau - Shuai K Shuai K Fau - Herschman HR Herschman Hr Fau - David M. Arginine methylation of STAT1 modulates IFNalpha/beta-induced transcription. Cell. (2001) 104(5):731–41. doi: 10.1016/s0092-8674(01)00269-0 11257227

[95] Nobori T Takabayashi K Tran P Orvis L Batova A Yu AL. Genomic cloning of methylthioadenosine phosphorylase: a purine metabolic enzyme deficient in multiple different cancers. Proc Natl Acad Sci U S A (1996) 93(12):6203–8. doi: 10.1073/pnas.93.12.6203 PMC39214 8650244

[96] Gao J Shi LZ Zhao H Chen J Xiong L He Q. Loss of IFN-gamma pathway genes in tumor cells as a mechanism of resistance to anti-CTLA-4 therapy. Cell (2016) 167(2):397–404.e9. doi: 10.1016/j.cell.2016.08.069 27667683 PMC5088716

[97] Kao HF Liao BC Huang YL Huang HC Chen CN Chen TC. Afatinib and pembrolizumab for recurrent or metastatic head and neck squamous cell carcinoma (ALPHA study): A phase II study with biomarker analysis. Clin Cancer Res (2022) 28(8):1560–71. doi: 10.1158/1078-0432.CCR-21-3025 PMC9306266 35046059

